# Kinetic Modifications of C_4_ PEPC Are Qualitatively Convergent, but Larger in *Panicum* Than in *Flaveria*

**DOI:** 10.3389/fpls.2020.01014

**Published:** 2020-07-03

**Authors:** Nicholas R. Moody, Pascal-Antoine Christin, James D. Reid

**Affiliations:** ^1^ Department of Chemistry, University of Sheffield, Sheffield, United Kingdom; ^2^ Department of Animal and Plant Sciences, University of Sheffield, Sheffield, United Kingdom

**Keywords:** C_4_ photosynthesis, carbon fixation, enzyme evolution, feedback inhibition, kinetics, phosphoenolpyruvate carboxylase

## Abstract

C_4_ photosynthesis results from a set of anatomical features and biochemical components that act together to concentrate CO_2_ within the leaf and boost productivity. This complex trait evolved independently many times, resulting in various realizations of the phenotype, but in all C_4_ plants the primary fixation of atmospheric carbon is catalyzed by phosphoenolpyruvate carboxylase. Comparisons of C_4_ and non-C_4_ PEPC from a few closely related species suggested that the enzyme was modified to meet the demands of the C_4_ cycle. However, very few C_4_ groups have been investigated, hampering general conclusions. To test the hypothesis that distant C_4_ lineages underwent convergent biochemical changes, we compare the kinetic variation between C_4_ and non-C_4_ PEPC from a previously assessed young lineage (*Flaveria*, Asteraceae) with those from an older lineage found within the distantly related grass family (*Panicum*). Despite the evolutionary distance, the kinetic changes between the non-C_4_ and C_4_ PEPC are qualitatively similar, with a decrease in sensitivity for inhibitors, an increased specificity (*k*_cat_/*K*_m_) for bicarbonate, and a decreased specificity (*k*_cat_/*K*_m_) for PEP. The differences are more pronounced in the older lineage *Panicum*, which might indicate that optimization of PEPC for the C_4_ context increases with evolutionary time.

## Introduction

C_4_ photosynthesis is a CO_2_-concentrating mechanism that boosts productivity in tropical conditions (Atkinson et al., 2016). The higher efficiency of C_4_ plants results from the increased concentration of CO_2_ around ribulose-1,5-bisphosphate carboxylase oxygenase (Rubisco), the entry enzyme of the Calvin–Benson cycle (Sage et al., 2012). Rubisco has a tendency to confuse CO_2_ and O_2_ (Tcherkez et al., 2006). The reaction of O_2_ produces compounds that need to be recycled in the energetically costly photorespiration pathway (Nisbet et al., 2007). In C_3_ plants, Rubisco is in direct contact with atmospheric gases, and photorespiration can become consequential in conditions that decrease the relative concentration of CO_2_, including high temperature, aridity and salinity (Ehleringer and Bjorkman, 1977; Skillman, 2007). C_4_ plants tackle this problem by segregating primary carbon fixation from the enzyme Rubisco into two cell types (Hatch, 1987; Sage, 2004; Sage et al., 2012). In C_4_ plants, atmospheric CO_2_ in the form of bicarbonate is initially fixed by the enzyme phosphoenolpyruvate carboxylase (PEPC) (Hatch, 1987). PEPC produces the four-carbon acid oxaloacetate, which is rapidly converted into the more stable four-carbon acids malate and/or aspartate (Bräutigam et al., 2014). These acids are shuttled to a cell isolated from the atmosphere in which Rubisco is localized, and CO_2_ is released. This biochemical pumping of CO_2_ leads to an increase of the relative concentration of CO_2_ by a factor of 10 when compared to a non-C_4_ cell, and a dramatic increase of photosynthetic efficiency at high temperature (Ehleringer and Bjorkman, 1977; von Caemmerer and Furbank, 2003; Sage, 2004; Sage et al., 2012).

The C_4_ photosynthetic mechanism is a classic example of convergent evolution, having evolved more than 60 times independently in various groups of flowering plants (Sage et al., 2011). As all known C_4_ enzymes exist in C_3_ plants, the evolution of C_4_ photosynthesis involved the co-option of genes and proteins essential for the cycle followed by adaption of their expression levels and, at least in some cases, their kinetic properties (Blasing et al., 2002; Tausta et al., 2002; Ghannoum et al., 2005; Aubry et al., 2011; Christin et al., 2013; Heckmann et al., 2013; Kulahoglu et al., 2014; Huang et al., 2017; Moreno-Villena et al., 2018; Alvarez et al., 2019; Niklaus and Kelly, 2019). In particular, the transcript level, enzyme abundance and activity of PEPC are massively increased in all C_4_ lineages screened so far (Engelmann et al., 2003; Marshall et al., 2007; Bräutigam et al., 2014; Christin et al., 2015; Moreno-Villena et al., 2018). In contrast, the kinetic behavior of the PEPC enzyme has received less attention and has been investigated mainly in a few systems of eudicot plants that contain closely related C_4_ and non-C_4_ species, such as the *Flaveria* genus [Asteraceae, (McKown et al., 2005)]. In *Flaveria*, the C_4_ PEPC has a ten-fold lower specificity for phosphoenolpyruvate (PEP), an increased sensitivity to activators such as glucose-6-phosphate, and a decreased sensitivity to feedback inhibition from malate and aspartate (Svensson et al., 1997; Engelmann et al., 2003; Svensson et al., 2003; Paulus et al., 2013a; DiMario and Cousins, 2018). Comparison of PEPCs from C_3_ to C_4_ intermediate species in *Flaveria* further suggested that C_4_ properties of the enzyme were gradually acquired during the diversification of the genus (Engelmann et al., 2003). Investigations of PEPC in Amaranthaceae, a distantly related family of eudicots that contains multiple C_4_ origins, have shown that PEP specificity evolved convergently in the two groups of C_4_ eudicots (Gowik et al., 2006). In contrast, kinetics of PEPC from grasses (Poaceae), the group that contains the largest number of C_4_ species, and the most productive and ecologically successful ones (Cerling et al., 1997; Osborne and Beerling, 2006; Sage et al., 2011), remain poorly known. Indeed, previous investigations of PEPC from grass species have used whole leaf preparations, which report on the behavior of mixtures of isoforms and not on well defined, pure enzymes (Huber and Edwards, 1975; Holaday and Black, 1981). PEPC isoforms are encoded by a multi-gene family, with at least six highly divergent gene lineages in most grasses (Christin et al., 2007). The kinetic behaviors have been compared among distant grass paralogs (Dong et al., 1998), but comparisons of closely related C_4_ and non-C_4_ orthologs in the family are missing.

According to molecular dating, the origins of C_4_ photosynthesis are spread throughout the last 35 million years (Christin et al., 2008; Christin et al., 2011). The genus *Flaveria* represents one of the most recent C_4_ origins, its different photosynthetic types having diverged in the last 3 million years, with the emergence of fully C_4_ plants 1–2 million years ago (Christin et al., 2011). While old C_4_ groups exist in eudicots, the previously investigated *Alternanthera* (Gowik et al., 2006) is only slightly older than *Flaveria*, having evolved the C_4_ trait 5–10 million years ago (Christin et al., 2011). With more than 22 C_4_ origins spanning a recent past up to 35 million years ago, the grass family contains the oldest and largest C_4_ lineages (Christin et al., 2008; Christin et al., 2011). In terms of C_4_ PEPC evolution, grasses and eudicots co-opted different genes (Christin et al., 2015). Genes encoding C_4_-specific PEPC evolved under positive selection in several C_4_ groups, but the identity and quantity of fixed amino acid changes varies among families (Besnard et al., 2009; Rosnow et al., 2015). In particular, more of these amino acid changes are observed among grasses than in *Flaveria* (Christin et al., 2007), which might result from the longer divergence between the photosynthetic types. Alternatively, the genes co-opted for C_4_ photosynthesis in grasses might have been less fit for the C_4_ function, requiring therefore more adaptive changes (Christin et al., 2010). Testing these hypotheses requires generating kinetic data for orthologous non-C_4_ and C_4_ PEPC genes from grasses. The PEPCs from *Flaveria* are well-studied (Svensson et al., 1997; Svensson et al., 2003; Paulus et al., 2013a; DiMario and Cousins, 2018) and make an excellent starting point for a detailed comparison with other non-characterized PEPCs.

In this work, we characterize the enzymes encoded by orthologous non-C_4_ and C_4_ genes from two grass species belonging to the same tribe (the C_4_ *Panicum queenslandicum* and the C_3_ *Panicum pygmaeum* from the tribe Paniceae) and compare them to non-C_4_ and C_4_ PEPC from *Flaveria* to test the hypotheses that (i) despite very different starting points, qualitatively similar changes happened in C_4_ PEPC from *Flaveria* and grasses, and (ii) the kinetic changes differ more between C_4_ and non-C_4_ PEPC in grasses than in *Flaveria* due to an expanded period of adaptive evolution. We describe the changes in specificity for both substrates (bicarbonate and PEP) as well as the nature of inhibition by aspartate and malate. Overall, out work sheds new light on the impacts of evolutionary time and distance on the convergent evolution of enzyme kinetics.

## Materials and Methods

Unless otherwise stated, reagents and components were from Sigma, protein purification equipment was from GE Healthcare and both enzymes for cloning and *E. coli* strains were from NEB.

### DNA Preparation

Genes that encode the *Flaveria trinervia* PEPC gene and the *Flaveria pringlei* PEPC gene in the pTrc 99A plasmid were provided by Peter Westhoff (Dusseldorf). The PEPC genes were sub cloned into the pET-1B His6 TEV LIC vector plasmid, provided by Scott Gradia (Berkley; Addgene plasmid #29653). Genes were sub cloned using the ligation independent cloning method with Q5 DNA polymerase and T4 DNA polymerase (NEB). Cloned plasmids were isolated using a Miniprep DNA kit (Qiagen). Plasmids were Sanger sequenced to confirm the sequence identity (GATC Biotech).

Leaf samples were collected from *P. queenslandicum* at midday in full daylight and flash frozen in liquid nitrogen. Leaf samples were homogenized with a pestle and mortar in liquid nitrogen. RNA was extracted from ground leaves using the RNeasy Kit (Qiagen). Libraries of cDNA were generated with SuperScript II Reverse Transcriptase (Thermo Fischer Scientific). The PEPC from *P. queenslandicum* was amplified using the primers PquFor1B and PquRev1B (**Supplementary Table 1**), and Q5 polymerase. The amplified gene was Sanger sequenced (GATC Biotech) with the PCR primers and with the primers Pqu_1323_Seq_For and Pqu_1752_Seq_Rev (Primers synthesized by Sigma, summarized in **Supplementary Table 1**). The gene was then cloned into the pET-1B His6 TEV LIC vector plasmid as above.

Because non-C_4_ PEPC from C_4_ grasses generally represent distant paralogs resulting from ancient duplications that predate the origin of the family (Christin et al., 2007), the most closely related non-C_4_ PEPC are in most cases those from related C_3_ species. We consequently selected a gene from a C_3_ species from the same tribe as *P. queenslandicum*. The sequence for PEPC from *P. pygmeaum* has been previously obtained *via* leaf transcriptome sequencing (Dunning et al., 2017). The sequence was codon optimized for expression in *E. coli* and synthesized by GenArt Gene Synthesis in the pTRCC Plasmid. The synthesized gene was sub-cloned into the pET-1B His6 TEV LIC plasmid and verified by Sanger sequencing.

### Protein Expression

For protein expression the BL2*1λ* (DE3) strain of *E. coli* (NEB) was used. Chemically competent *E. coli* cells were transformed with each of the plasmids. Eight liters of cultures were grown in LB media at 37°C to OD_600_ 0.8. Cultures were cooled to 4°C for 1 h prior to recombinant protein induction with 0.5 mM IPTG (Fischer). Cultures were then incubated at 18°C for 18 h. Cells were harvested by centrifugation at 5,400×*g* for 25 min and stored at −80°C.

### Protein Purification

Cells were suspended in IMAC buffer (25 mM Tris, 0.5 M NaCl, 0.3 M glycerol, 20 mM imidazole (Acros Scientific)), 10 ml per 2 L of culture with 50 µl of 50 mgml^−1^ DNase I and 100 µl of 100 mgml^−1^ Pefabloc. Cells were passed twice through a cell disruptor (Constant Systems) before centrifugation at 276,000×*g* for 40 min. The supernatant was passed through a 0.45 µm pore filter (Elkay Labs.). PEPC was separated from soluble protein with a prepacked 1 ml nickel affinity column using an ÄKTA Pure 25 L Chromatography System. The loaded column was washed with 50 column volumes of IMAC buffer, then 50 column volumes of IMAC buffer containing 150 mM imidazole. Pure PEPC was eluted with 10 column volumes of IMAC buffer containing 400 mM imidazole.

Protein eluted from IMAC purification was loaded onto a Sephadex G50 desalting column (Amersham Biosciences) and rebuffered in storage buffer (20 mM Tris, 5% v/v glycerol, 150 mM KCl, 1 mM DTT (AnaSpec. Inc.)). Protein was aliquoted and frozen at −80°C until use.

### Enzyme Quantification

PEPC enzyme concentration was quantified by absorption at 280 nm. Enzyme extinction coefficients were calculated using the ExPASy protein parameter tool and corrected by determining the absorbance of the protein denatured in 6 M guanidine hydrochloride (Gill and von Hippel, 1989). The extinction coefficients for the *F. trinervia*, *F. pringlei*, *P. queenslandicum* and *P. pygmaeum* PEPC were 120,480 M^−1^cm^−1^, 117,030 M^−1^cm^−1^, 105,810 M^−1^cm^−1^ and 111,510 M^−1^cm^−1^, respectively. Gel based protein quantification was not used.

Protein samples were analyzed for purity using SDS PAGE analysis. Samples of cell lysate or pure protein (25 µg or 5 µg protein respectively; BCA assay from Pierce) were denatured in 2 × SDS PAGE loading dye (200 mM Tris HCl pH 6.8, 2% SDS, 20% Glycerol, 0.01% Bromophenol blue (BDH Laboratory Supplies) and 7% β-mercaptoethanol). Protein was loaded onto an 8% acrylamide SDS gel with 2 µl of Blue Prestained Protein Standard Broad Range (11–190 kDa) (NEB). Gels were run for 50 min at 200 V with 1 × Tris/Glycine/SDS running buffer (Geneflow). Gels were stained with InstantBlue (Expedeon) and imaged with a ChemiDoc MP (BioRad).

### Enzyme Assays

PEPC activity was measured spectroscopically at 340 nm by coupling to NADH-malate dehydrogenase. Assays with a high fixed concentration of bicarbonate were observed using a FLUOstar plate reader (BMG Labtech) through a 340 nm ± 5 nm filter in absorbance mode. These assays were conducted in a reaction volume of 150 µl at 25°C. A typical reaction mixture contained 50 mM Tricine.KOH pH 8.0, 10 mM MgCl_2_ (Fluka), 5 mM KHCO_3_, 0.2 mM NADH (Fischer) and 0.1 U µl^−1^ malate dehydrogenase. Assays were initiated with the addition of PEPC enzyme. Rates were calculated with a NADH calibration curve; this method takes account of the short pathlength in microtiter plates.

Assays at a range of bicarbonate concentrations were observed with a Cary spectrophotometer (Agilent Technologies) in the same reaction buffer, in a total reaction volume of 600 µl. Initial rates were calculated using the Cary analysis software. To remove background bicarbonate, the water and tricine buffer were sparged with nitrogen for 18 h prior to use in assays. These assays were constructed under a nitrogen flow and performed in a sealed cuvette. The reaction was initiated with the addition of 50 nM PEPC, delivered with a gastight syringe (Hamilton). Bicarbonate concentrations were controlled with the addition of freshly prepared potassium bicarbonate.

The background bicarbonate was determined using an endpoint assay with no potassium bicarbonate (30 min). This procedure determines the total concentration of dissolved and hydrated CO_2_, (i.e. CO_2__(aq)_, H_2_CO_3_, HCO_3_^−^ and CO_3_^2−^), at this pH over 97% is in the form of bicarbonate. Reported bicarbonate concentrations are the sum of the background and the added bicarbonate.

### Data Analysis

Kinetic parameters were evaluated by non-linear regression analysis in Igor Pro (Version 7.0.8.1; Wavemetrics Inc., Lake Oswego, Oregon). In all cases, the enzyme was assumed to be fully active. Primary plots were analyzed using Equation (1).

(Equation 1) $$\frac{v_{i}}{{\left[E\right]}_{T}}=\frac{k_{cat}^{app}[S]}{K_{M}^{app}+[S]}$$

Analysis of secondary plots (*i.e.* of $k_{cat}^{app}$ or $k_{cat}^{app}/k_{m}^{app}$ vs [PEP]) with Equation (2) allowed determination of the steady-state kinetic parameters, $k_{cat}/K_{m}^{HCO3-}$ and $k_{cat}/K_{m}^{PEP},\;K_{i}^{PEP}$ and *k*_cat_.

(Equation 2) $$k=\frac{k^{app}[S]}{K+[S]}$$

Where *k* and *k* ^app^ are the true and apparent values of *k*_cat_ or $k_{cat}/K_{m}^{HCO3-}$ and *K* is $K_{m}^{PEP}$ or $K_{i}^{PEP}$.

In the case of inhibition data, secondary plots were analyzed using Equation (3), where in the case of competitive inhibition *k* ^app^ is $k_{cat}^{app}/k_{m}^{app}$ and *K*_i_ is the competitive inhibition constant *K*_ic_ or in the case of non-competitive inhibition *k* ^app^ is $k_{cat}^{app}$ and *K*_i_ is the non-competitive inhibition constant *K*_iu_.

The non-competitive inhibition constant (*K*_iu_) was determined by the secondary plot of $k_{cat}^{app}$ against inhibitor concentration. The competitive inhibition constant (*K*_ic_) was determined by the secondary plot of $k_{cat}^{app}/k_{m}^{app}$ against inhibitor concentration.

(Equation 3) $$k^{app}=\frac{k}{1+\frac{\left[I\right]}{K_{i}}}$$

All data points shown on plots of initial rate against substrate concentration are individual measurements. Standard errors are provided for every parameter estimate. In secondary plots of apparent kinetic parameters against substrate or inhibitor concentration the standard error of those parameter estimates are shown. These standard errors are provided directly by the nonlinear regression analysis routine implemented within Igor Pro.

## Results

### DNA Cloning and Protein Purification

Four PEPC isoforms were characterized. In grasses, the C_4_ and non-C_4_ forms of *ppc-1P3* genes were isolated from the C_4_ *P. queenslandicum* and synthesized based on the sequence of the C_3_ species *P. pygmaeum*, respectively. The cloned genes were 962 and 969 codons long, respectively. They have an 86.2% identity in amino acids and a 93.2% similarity, including on the two positions that have been linked in C_4_ *Flaveria* to $K_{m}^{PEP}$ and decreased inhibition (positions 774 and 884, respectively; Blasing et al., 2000; DiMario and Cousins, 2018). In *Flaveria*, the C_4_ and non-C_4_ *ppc-1E2* genes corresponding to the C_4_ *F. trinervia* and the C_3_ species *F. pringlei* were analyzed [ppcA as described in Svensson et al. (1997)]. The two genes are both 967 codons long, with a 94.7% identity and a 97.5% similarity. The orthologous relationships between these pairs of genes were confirmed by phylogenetic analyses (**Supplementary Figure 1**).

All four genes were prepared in vectors for over-expression in *E. coli* with an N-terminal His tag. In all cases, expressed protein was purified to >95% purity as assessed by SDS PAGE with a single immobilized metal column (**Supplementary Figure 2**).

### The Presence of an N-Terminal His_6_ Tag Does Not Affect Activity

Assays at saturating bicarbonate and variable concentrations of PEP (**Supplementary Figure 3**) showed that both His tagged *Flaveria* PEPCs behaved similarly to untagged proteins previously described (Svensson et al., 1997; Blasing et al., 2000; Jacobs et al., 2008). Specifically, at pH 8.0, 10 mM MgCl_2_, 10 mM KHCO_3_, coupled to malate dehydrogenase, the His_6_-PEPC from *F. trinervia* catalyses the formation of oxaloacetate with a $K_{m}^{PEP}$ of 0.61 ± 0.05 mM and a *k*_cat_ of 47.99 ± 1.22 s^−1^. Literature values are $K_{m}^{PEP}$ ranging from 0.278 to 0.652 mM and *V*_max_ of 29 U mg^−1^, allowing for the different protein concentration, our *k*_cat_ would be equivalent to a *V*_max_ of 25.56 U mg^−1^ (Svensson et al., 1997; Blasing et al., 2000). Under the same conditions, the His_6_-PEPC from *F. pringlei* catalyses the formation of oxaloacetate with a $K_{m}^{PEP}$ of 0.05 ± 0.01 mM and a *k*_cat_ of 52.65 ± 1.37 s^−1^; literature values are $K_{m}^{PEP}$ ranging from 0.029 to 0.061 mM and *V*_max_ of 27 U mg^−1^, and allowing for the different protein concentration, our *k*_cat_ would be equivalent to a *V*_max_ of 28.02 U mg^−1^ (Svensson et al., 1997; Blasing et al., 2000). This confirms previous reports (Paulus et al., 2013a) that the presence of an N-terminal poly-histidine tag does not adversely affect the activity of these proteins.

### Kinetic Analyses Demonstrate That the C_4_ Enzyme Forms Show a Lower *k_cat_/K_m_* Towards PEP and a Higher *k_cat_/K_m_* to Bicarbonate Than the Related Non-C_4_ Forms

**Figure 1:**
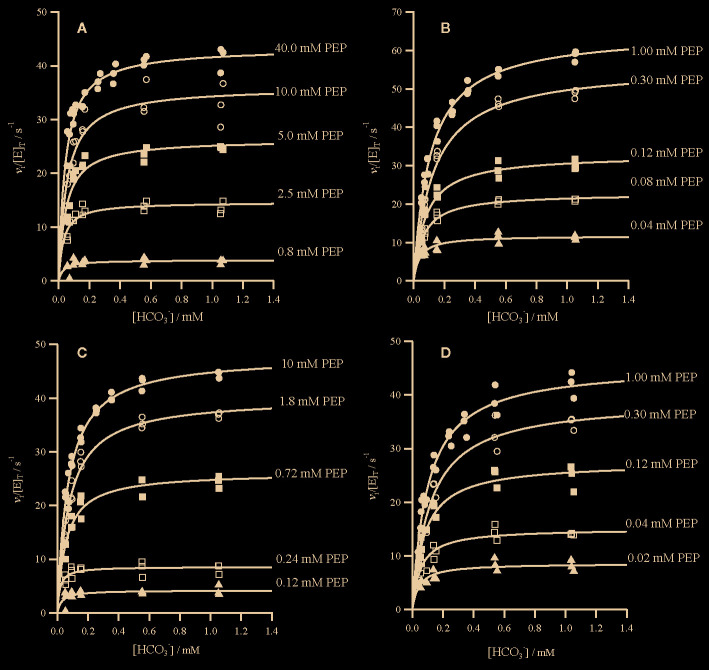
Initial rates of oxaloacetate formation catalyzed by PEPC. Assay conditions were 50 mM Tricine.KOH pH 8.0, 10 mM MgCl_2_, 0.2 mM NADH, 0.01 Uμl^−1^ malate dehydrogenase and 50 nM PEPC at 25°C, the concentration of PEP also varied as shown. Assays were repeated (*n* = 3) at each concentration. Individual data points are shown for the following PEPC **(A)** *Panicum queenslandicum* **(B)** *Panicum pygmaeum* **(C)** *Flaveria trinervia* and **(D)** *Flaveria pringlei* PEPC. Kinetic parameters are summarized in **Table 1**.

The specificity for bicarbonate of all four enzymes was determined using a gas-tight assay system. Background bicarbonate was reduced to *ca.* 50 µM by sparging with nitrogen gas. Assays were performed at five PEP concentrations, while varying the concentration of bicarbonate (**Figure 1**). The analysis of secondary plots (**Supplementary Figures 4** and **5**) provided estimates of *k*_cat_ and the specificity constant, *k*_cat_/*K*_m_, for both substrates (**Table 1**).

**Table 1:** Summary of kinetic parameters of PEPC found in this study.

PEPC Species	*k*_cat_ (s^-1^)	$K_{m}^{PEP}$ (mM)	$K_{i}^{PEP}$ (mM)	$k_{cat}/K_{m}^{PEP}$ (M^−1^s^−1^)	$K_{m}^{HCO3-}$ (mM)	$k_{cat}/K_{m}^{HCO3-}$ (M^−1^s^−1^)
*Panicum queenslandicum* (C_4_)	46.96 ± 1.71	4.17 ± 0.30	4.39 ± 1.10	0.01× 10^6^ ± 0.11 × 10^4^	0.04 ± 0.02	1.09 × 10^6^ ± 8.88 × 10^4^
*Panicum pygmaeum* (C_3_)	65.59 ± 1.74	0.17 ± 0.05	0.05 ± 0.01	0.50 × 10^6^ ± 2.44 × 10^4^	0.12 ± 0.02	0.60 × 10^6^ ± 2.93 × 10^4^
*Flaveria trinervia* (C_4_)	47.99 ± 1.21	0.60 ± 0.05	0.40 ± 0.13	0.08 × 10^6^ ± 0.54 × 10^4^	0.07 ± 0.01	0.69 × 10^6^ ± 4.17 × 10^4^
*Flaveria pringlei* (C_3_)	52.65 ± 1.37	0.06 ± 0.01	0.02 ± 0.01	0.94 × 10^6^ ± 8.49 × 10^4^	0.10 ± 0.01	0.44 × 10^6^ ± 2.17 × 10^4^

Standard errors are given, based on fitted theoretical curves.

The specificity for bicarbonate (*k*_cat_/*K*_m_) of the C_4_ *P. queenslandicum* PEPC is 1.09 × 10^6^ M^−1^s^−1^, almost twice as large as that of the non-C_4_ *P. pygmaeum* enzyme (**Table 1**). The specificity of this non-C_4_ enzyme is comparable to that of the C_4_ PEPC of *Flaveria* at 0.69 × 10^6^ M^−1^s^−1^ (**Table 1**), which again is slightly higher than that of the *Flaveria* non-C_4_ PEPC (**Table 1**). In both cases the specificity constant for PEP is smaller in the C_4_ form of the enzyme (**Table 1**). In terms of bicarbonate *K*_m_ values these are within the range previously reported for C_4_ and non-C_4_ plant PEPC isoforms in work with reasonably careful control of background bicarbonate (O'Leary, 1982; Bauwe, 1986; Janc et al., 1992; Dong et al., 1998; DiMario and Cousins, 2018).

### Both C_4_ PEPC Enzymes Are Less Sensitive to the Inhibitors Malate and Aspartate at Any Concentration of PEP

For both non-C_4_ and C_4_ enzymes, we investigated inhibition by the two feedback inhibitors, malate (**Supplementary Figure 6**) and aspartate (**Supplementary Figure 7**) across a range of PEP concentrations. These two structurally related inhibitors show different kinetic characteristics; unlike aspartate, malate remains an inhibitor at saturating concentration of PEP (**Table 2**).

**Table 2:** Summary of inhibition parameters of PEPC found in this study.

PEPC Species	$K_{ic}^{Malate}$ (mM)	$K_{iu}^{Malate}$ (mM)	$K_{ic}^{Aspartate}$ (mM)
*Panicum queenslandicum* (C_4_)	7.51 ± 1.17	146.08 ± 20.40	49.44 ± 7.86
*Panicum pygmaeum* (C_3_)	0.52 ± 0.22	31.23 ± 0.65	2.27 ± 0.02
*Flaveria trinervia* (C_4_)	10.96 ± 1.55	40.72 ± 4.59	40.02 ± 6.49
*Flaveria pringlei* (C_3_)	2.14 ± 0.62	4.56 ± 1.72	4.13 ± 0.60

Standard errors are given, based on fitted theoretical curves.

**Figure 2:**
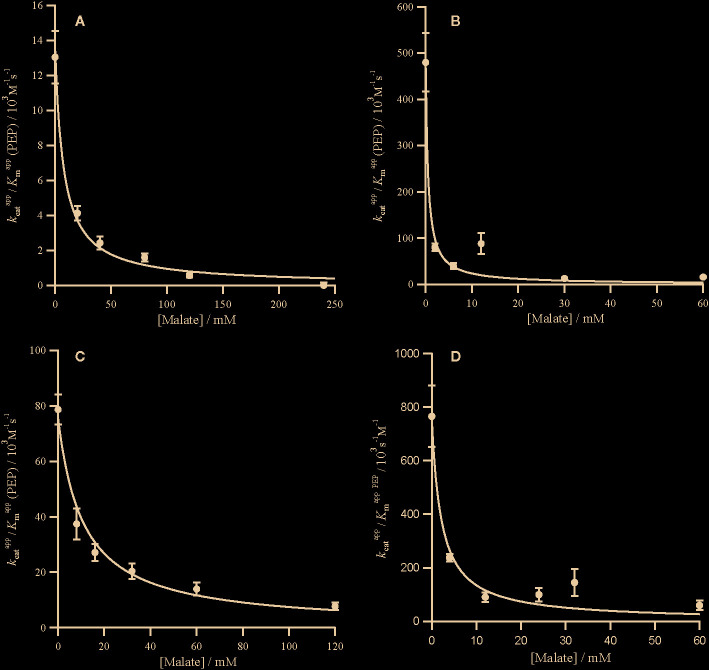
Competitive inhibition of PEPC by malate. Markers represent $k_{cat}^{app}/K_{m}^{appPEP}$ from assays in the presence of malate (**Supplementary Figure 6**) and error bars represent the standard errors. $k_{cat}^{app}/K_{m}^{appPEP}$ against malate concentration with inhibition curves characterized by Equation (3) and a *K*_ic_ for the following PEPC **(A)** *Panicum queenslandicum* ($K_{ic}^{Malate}$= 7.51 ± 1.17 mM), **(B)** *Panicum pygmaeum* ($K_{ic}^{Malate}$= 0.52 ± 0.22 mM), **(C)** *Flaveria trinervia* ($K_{ic}^{Malate}$= 10.96 ± 1.55 mM), and **(D)** *Flaveria pringlei* ($K_{ic}^{Malate}$= 2.14 ± 0.62 mM). Inhibition parameters are summarized in **Table 2**.

**Figure 3:**
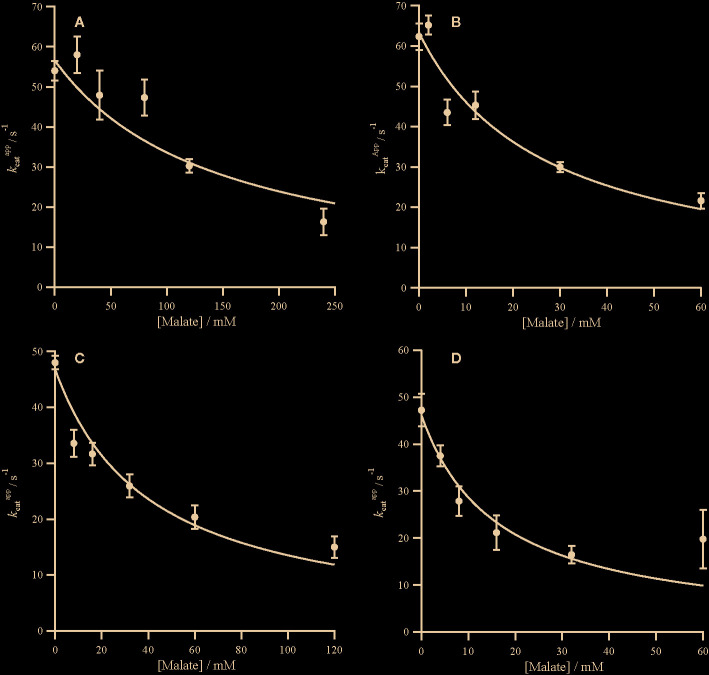
Non-competitive inhibition of PEPC by malate. Markers represent the $k_{cat}^{app}$ from assays in the presence of malate (**Supplementary Figure 6**) and error bars represent the standard errors. $k_{cat}^{app}$ against malate concentration with inhibition curves characterized by Equation (3) and a *K*_iu_ for the following PEPC **(A)** *Panicum queenslandicum (* $K_{iu}^{Malate}$= 146.08 ± 20.40 mM), **(B)** *Panicum pygmaeum* ($K_{iu}^{Malate}$= 31.23 ± 0.65 mM), **(C)** *Flaveria trinervia* ($K_{iu}^{Malate}$= 40.72 ± 4.59 mM) and **(D)** *Flaveria pringlei* ($K_{iu}^{Malate}$ = 4.56 ± 1.72 mM). Inhibition parameters are summarized in **Table 2**.

**Figure 4:**
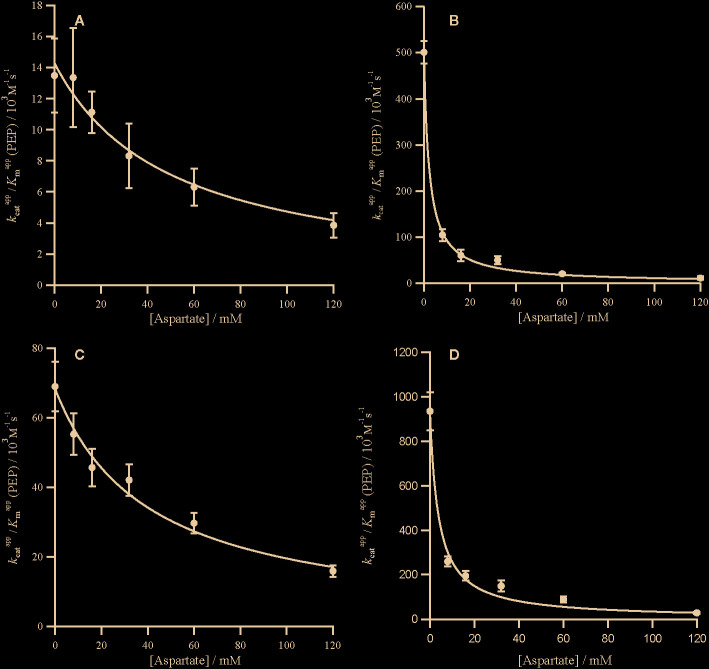
Competitive inhibition of PRPC by aspartate. Markers represent the $k_{cat}^{app}/K_{m}^{appPEP}$ from assays in the presence of aspartate (**Supplementary Figure 7**) and error bars represent the standard errors. $k_{cat}^{app}/K_{m}^{appPEP}$ against aspartate concentration with inhibition curves characterized by Equation (3) and a *K*_ic_ for the following PEPC **(A)** *Panicum queenslandicum* ($K_{ic}^{Aspartate}$= 49.44 ± 7.86 mM), **(B)** *Panicum pygmaeum* ($K_{ic}^{Aspartate}$= 2.27 ± 0.02 mM), **(C)** *Flaveria trinervia* ($K_{ic}^{Aspartate}$= 40.02 ± 6.49 mM) and **(D)** *Flaveria pringlei* ($K_{ic}^{Aspartate}$= 4.31 ± 0.60 mM). Inhibition parameters are summarized in **Table 2**.

The C_4_ cycle of *Flaveria* produces both malate and aspartate (Moore and Edwards, 1986; Meister et al., 1996), while *Panicum* species are expected to produce mainly aspartate around PEPC (Rao and Dixon, 2016). The two molecules have however been shown to inhibit PEPC in a variety of C_4_ species (Huber and Edwards, 1975). In our analyses, all the PEPC enzymes are inhibited by malate at both limiting and saturating concentrations of PEP, and malate is a mixed inhibitor (**Figures 2** and **3**). This mixed inhibition can be characterized by two inhibition constants; $K_{ic}^{Malate}$ at limiting PEP and $K_{iu}^{Malate}$ at saturating PEP. In all cases, $K_{ic}^{Malate}\mathrm{>>}K_{iu}^{Malate},$ which means that malate can be viewed as a predominantly competitive inhibitor. The two C_4_ forms of the enzyme are both less sensitive to malate than the two non-C_4_ forms (**Table 2**). Unlike malate, aspartate is solely a competitive inhibitor for all of these enzymes (**Figure 4**). Increasing concentrations of aspartate do not affect *k*_cat_ (**Supplementary Figure 8**). Once again, the two C_4_ forms of the enzyme are much less sensitive to aspartate than the two non-C_4_ forms (**Table 2**). Overall, our analyses indicate that the C_4_ forms are much less sensitive to both inhibitors, independently of the taxonomic group and C_4_ subtype, confirming previous reports (Huber and Edwards, 1975).

## Discussion

### Convergent Kinetic Changes Across C_4_ Flowering Plants

The non-C_4_ genes encoding the PEPC enzymes of the C_3_ plants *P. pygmaeum* and *F. pringlei* diverged about 150 million years ago and since then have accumulated numerous mutations and undergone multiple gene duplications (Christin et al., 2007; Christin et al., 2015). They share an 83.5% identity and a 91.2% similarity, and greater than 93% similarity with their respective C_4_ proteins. While the exact function of each non-C_4_ isoform is unknown, they are transcribed at similarly moderate levels (Moreno-Villena et al., 2018). Our investigation shows that the two non-C_4_ enzymes characterized here exhibit functionally similar kinetic characteristics, including high sensitivity to competitive inhibition by malate and aspartate and a similar sensitivity to bicarbonate. However, the two non-C_4_ isoforms differ in their $K_{m}^{PEP}$ which is three-fold lower in the *F. pringlei* enzyme (**Table 1**). While systematic screens of non-C_4_ PEPC are missing, those of *Alternanthera* and a distant root paralog from *Z. mays* have a similar $K_{m}^{PEP}$ to the *F. pringlei* enzyme (Dong et al., 1998; Gowik et al., 2006). These data suggest that despite hundreds of million years of divergence, non-C_4_ PEPC are generally associated with high sensitivity to inhibitors and low (<0.2 mM) *K*_m_ for both substrates. These properties are likely required for a tight regulation and fast response of isoforms involved in anaplerotic functions, where the concentrations of substrates and products are low.

In both *Flaveria* and *Panicum*, the C_4_ PEPC shows a markedly reduced sensitivity to both malate and aspartate as compared with the non-C_4_ ortholog (**Table 2**). This reduction in sensitivity, reported before in *Flaveria* (Blasing et al., 2002; Paulus et al., 2013b; DiMario and Cousins, 2018) and a variety of grasses from different C_4_ subtypes (Huber and Edwards, 1975), is observed at all concentrations of PEP (**Figures 2**–**4**). Our observations are thus consistent with the conclusion that the same selective pressures act in C_4_ eudicots and at least some grasses to decrease the sensitivity to the inhibitors malate and aspartate. In C_4_ plants the concentration of malate and aspartate are high, so this reduced sensitivity prevents PEPC being inhibited by downstream metabolites (Arrivault et al., 2017). The respective amounts of malate and aspartate vary among C_4_ species (Moore and Edwards, 1986; Meister et al., 1996; Rao and Dixon, 2016), and concerted reduction of inhibition by both species is consistent with them sharing a binding site (Paulus et al., 2013a).

The adaptation of PEPC to the demands of the C_4_ pathway involved qualitatively similar changes in substrate specificity between *Flaveria* and the grasses considered here (**Table 1**). In both cases the specificity for PEP decreases and the specificity for bicarbonate increases. The C_4_ form of *Zea mays*, an independent C_4_ origin within grasses, has an affinity for PEP that is similar to *P. queenslandicum* (Dong et al., 1998). In addition, changes of *K*_m_ for PEP in the same direction have been reported in *Alternanthera* (Gowik et al., 2006), suggesting that decreases in PEP *K*_m_ happened convergently across C_4_ origins. The functional value of these changes remains speculative and might be a side-effect of adaptation of other properties of the enzyme or a direct target of selection for tighter regulation when PEP concentrations are higher (Svensson et al., 2003). The differences in *K*_m_ for bicarbonate are less marked than those of PEP (**Table 1**). The *K*_m_ for bicarbonate parameter is much higher in the non-C_4_ root isoform from *Z. mays* (Dong et al., 1998), indicating it varies tremendously among non-C_4_ PEPC. Data from more species are needed to determine whether the qualitative convergence observed here between *Flaveria* and *Panicum* is universal, or depends on the co-opted gene or the details of the C_4_ phenotype (e.g. biochemical and anatomical subtypes). Indeed, the cellular concentration of bicarbonate depends on the action of the enzyme carbonic anhydrase, in addition to the cell pH, and it is thus possible that variation in these factors changes the adaptive value of bicarbonate affinity.

### The Differences in Enzyme Behavior Are Quantitatively More Important in *Panicum* Than in *Flaveria*

While differences in substrate specificity and sensitivity to inhibitors are qualitatively convergent between *Flaveria* and the two grasses considered here, they are more marked in the latter (**Table 1**). These quantitative differences might be linked to the contrast between the length of time spent as C_4_ in each lineage, from more than 16 million years for *P. queenslandicum* to less than three for *Flaveria* (Christin et al., 2008; Christin et al., 2011). The C_4_ PEPCs share a 76.5% identity and an 88.1% similarity. Indeed, the kinetic properties observed in the PEPC of extant taxa result from adaptive changes accumulated since the initial origin of C_4_ photosynthesis. According to current models, an initial C_4_ pathway can evolve *via* enzyme upregulation and limited modifications of the proteins (Sage et al., 2012; Heckmann et al., 2013; Dunning et al., 2019; Heyduk et al., 2019), as observed in C_3_–C_4_ intermediates (Svensson et al., 2003; Dunning et al., 2017). Once a C_4_ pathway is in place, selection will act to improve its efficiency (Heckmann et al., 2013), and variation among members of the same C_4_ lineage indicates that such process can take protracted periods of selection on novel mutations (Heyduk et al., 2019).

Because these are likely necessary for a function of PEPC in C_4_ cells with high concentrations of metabolites, we suggest that relaxed sensitivity to inhibitors happens early during the evolution of C_4_ PEPC. This hypothesis is supported by the fact that changes in sensitivity to inhibitors are observed in intermediates from *Flaveria* (Engelmann et al., 2003). It has moreover been shown that one single amino acid replacement is sufficient to generate a large decrease in sensitivity (Paulus et al., 2013a). The C_4_-specific residue at this site is observed in multiple C_4_ lineages of both grasses and eudicots (Paulus et al., 2013a; Paulus et al., 2013b), suggesting that a rapid decrease of inhibition is involved in many origins of C_4_ PEPC.

Other properties of C_4_-specific PEPC might represent secondary adaptations to the C_4_ context, which might happen either to strengthen the early trends or in response to other changes of the plant biochemical phenotype. Over time, sustained diversifying selection on C_4_ PEPC would have led to stronger differences between *P. queenslandicum* and the C_3_ grasses. This view is supported by the similar kinetic parameters between the C_4_ PEPC of *P. queenslandicum* and *Zea mays*, two grass lineages of similar age, as well as similar kinetic parameters observed between the C_4_ PEPC in *Alternanthera* and *Flaveria*, two comparatively young lineages (Christin et al., 2011). It is however possible that secondary PEPC adaptations vary among and maybe even within old C_4_ lineages, as different biochemical and anatomical C_4_ subtypes evolved. Data from more lineages are needed to test the hypothesis that such diversifying PEPC secondary adaptation happened.

The molecular basis of the C_4_ specific properties reported here are not well understood. Analysis of the evolution of the amino acid sequence of C_4_ PEPC has shown that at least 22 sites underwent positive selection in grasses and sedges (Christin et al., 2007). Of these sites, three are also observed in C_4_ *Flaveria* (Christin et al., 2007; Besnard et al., 2009). Some of these mutations have been shown to be responsible for key C_4_ specific kinetic properties. Of these, a mutation for alanine to serine at position 774 (*Flavaria* numbering) has been identified as an important determinant of the low specificity for PEP of the C_4_ form of the enzyme (Blasing et al., 2000); interestingly, the effect of this position on bicarbonate specificity depends on the rest of the sequence and concentrations of allosteric regulators (DiMario and Cousins, 2018). Additionally, a mutation at position 884 (*Flaveria* numbering), in the allosteric inhibitor binding site, has been shown to have a notable effect on the IC_50_ for malate. An arginine residue in this position, as seen in the non-C_4_ form of the enzyme is well placed to directly interact with the inhibitor, increasing the susceptibility to inhibition (Paulus et al., 2013a). These amino acid changes are observed in the C_4_ PEPC of *Panicum* but not its non-C_4_ ortholog, and presumably contribute to the kinetic differences between the two. The specific role of other grass mutations has yet to be identified, a task that will be complicated by the large amount of variation among grass PEPC and possible epistasy among sites. These factors make it difficult to associate specific kinetic changes with specific amino acid replacements. Here, we compared the characteristics of PEPC from old, diverse lineages; these efforts now need to be expanded to other C_4_ lineages, with the well characterized isoforms of *Flaveria* continuing to serve as a model to assess the effect of specific sites.

## Data Availability Statement

All datasets presented in this study are included in the article/**Supplementary Material**.

## Author Contributions

All authors contributed to the article and approved the submitted version. NM carried out all experimental work.

## Funding

P-AC is supported by a Royal Society University Research Fellowships (URF\R\180022), JR is supported by the Biotechnology and Biological Sciences Research Council (BBSRC, UK; award number BB/M000265/1). NM was supported by the Grantham Centre for Sustainable Futures.

## Conflict of Interest

The authors declare that the research was conducted in the absence of any commercial or financial relationships that could be construed as a potential conflict of interest.
