# Supplementary material for: Kinetic Modifications of C4 PEPC Are Qualitatively Convergent, but Larger in Panicum Than in Flaveria
Source: Front Plant Sci. 2020 Jul 3;11:1014. doi: 10.3389/fpls.2020.01014 (PMC7350407; doi:10.3389/fpls.2020.01014)
Supplement: Supplementary file 1 [file DataSheet_1.pdf]

Supplementary Material

1 Supplementary Figures and Tables

For more information on Supplementary Material and for details on the different file types accepted, please see here. Figures, tables, and images will be published under a Creative Commons CC-BY licence and permission must be obtained for use of copyrighted material from other sources (including re-published/adapted/modified/partial figures and images from the internet). It is the responsibility of the authors to acquire the licenses, to follow any citation instructions requested by third-party rights holders, and cover any supplementary charges.

1.1 Supplementary Figures

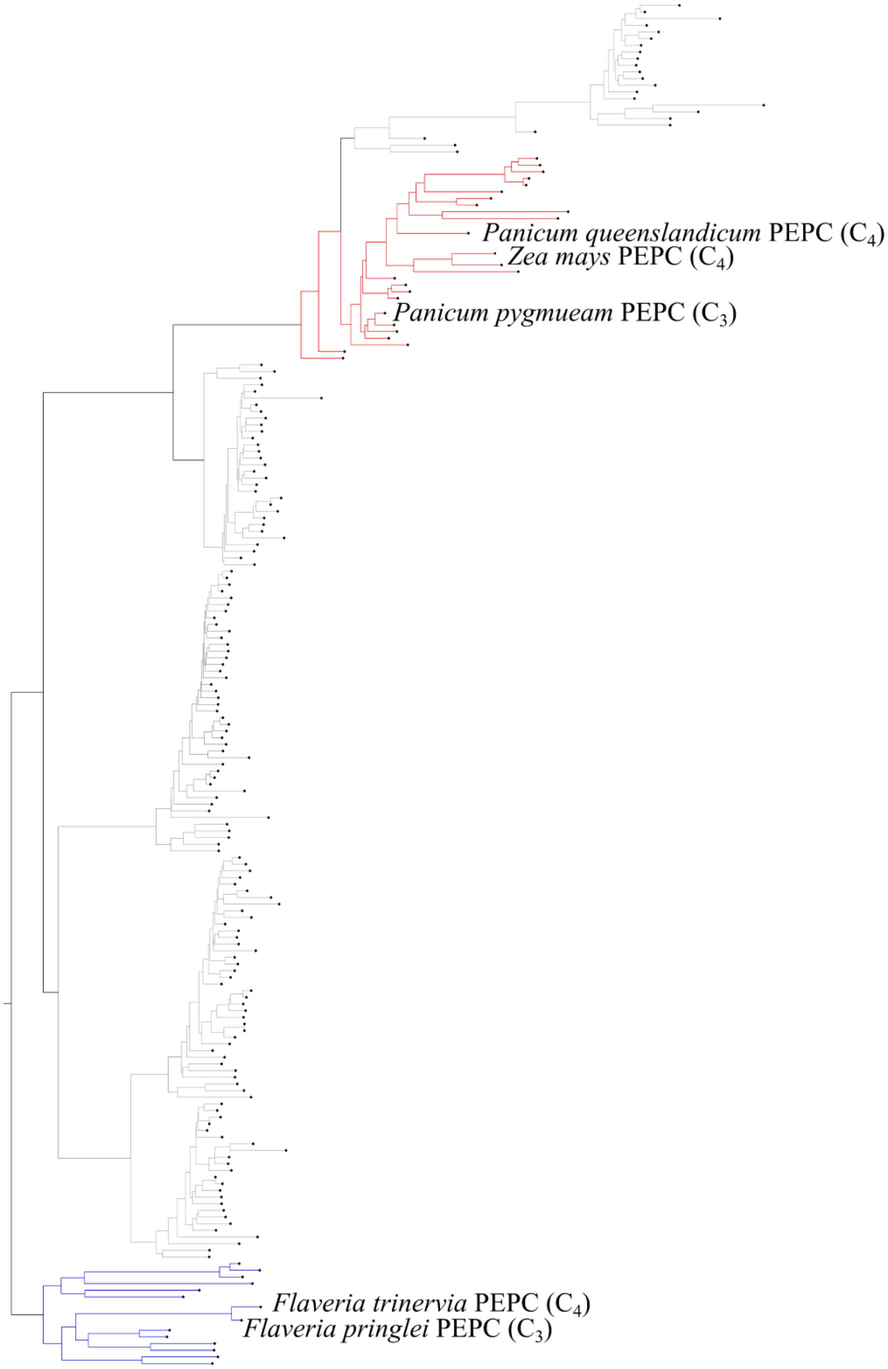

Supplementary Figure 1. Maximum likelihood tree of orthologous PEPC genes. In red are the ppc1P3 isoforms. In blue are the ppc1E2 isoforms. Sequences for *Zea mays* (C_4) taken from (Dong et al., 1998).

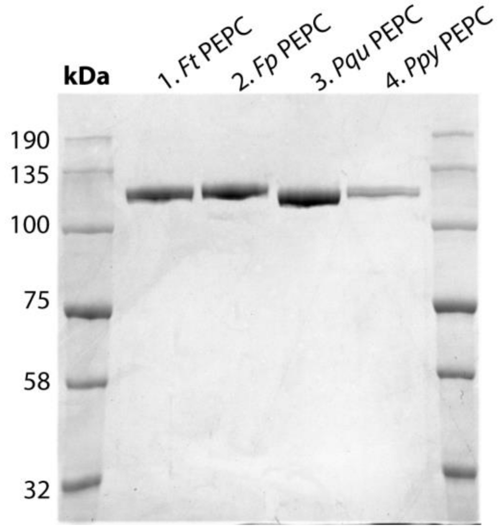

Supplementary Figure 2. SDS PAGE gel of purified PEPC Enzymes. 8 % acrylamide SDS PAGE analysis of PEPC proteins (5 µg by BCA assay) compared in this study. Gel visualised with instant blue. PEPC from 1. *Flaveria trinervia*, 2. *Flaveria pringlei*, 3. *Panicum queenslandicum* and 4. *Panicum pygmaeum* PEPC.

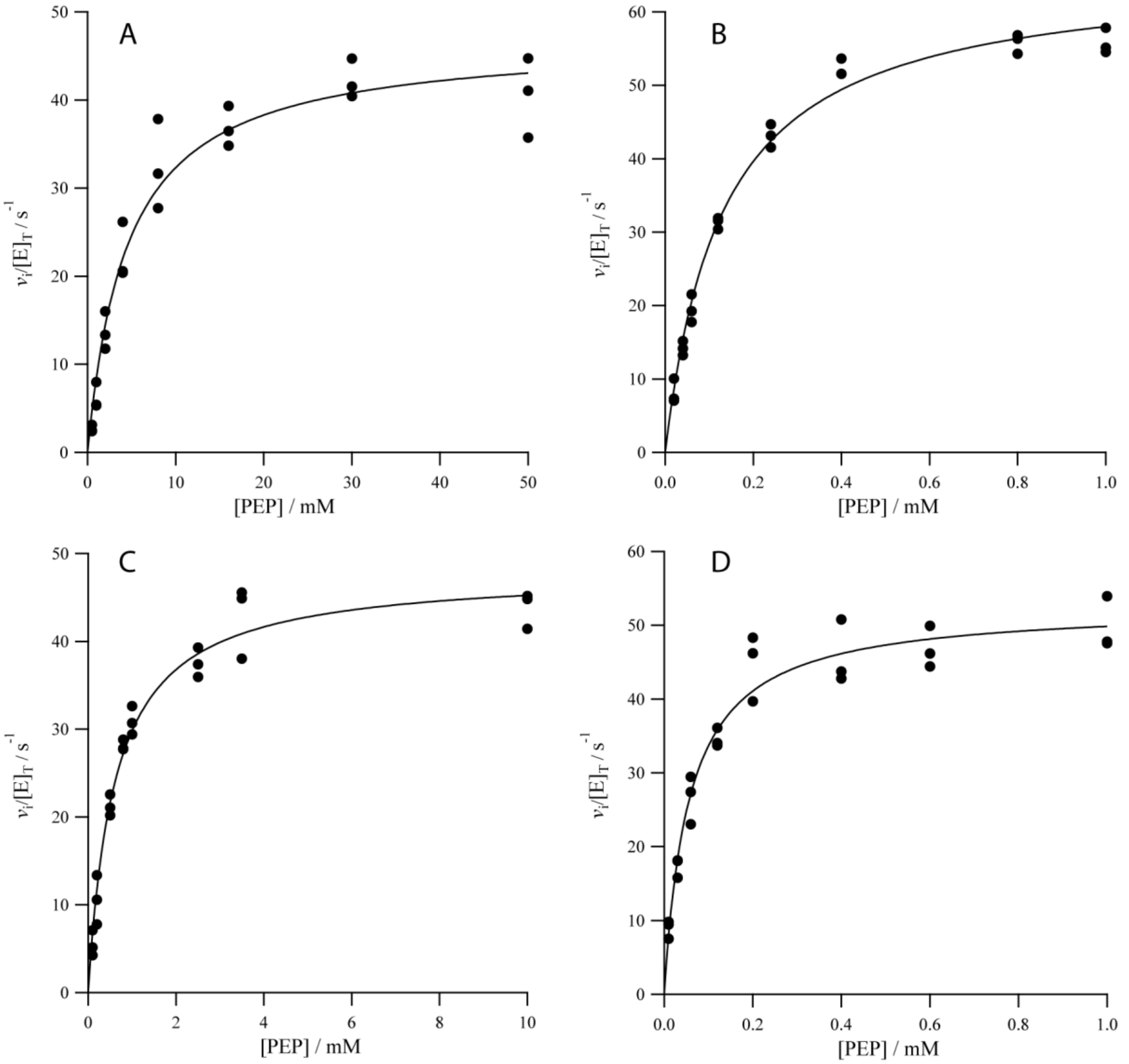

Supplementary Figure 3. **PEP Michaelis-Menten Assays (v_i vs PEP).** Assays conditions were 50 mM Tricine.KOH pH 8.0, 10 mM $MgCl_2$, 0.2 mM NADH, 0.01 Uμl^{-1} malate dehydrogenase, initiated by 10 nM PEPC. Assays were repeated (n = 3) for each concentration of PEP. Lines are described by equation 1. (A) Markers experimental data for *Panicum queenslandicum* PEPC, the line is characterised by the parameters k_{cat} = 46.96 ± 1.74 s^{-1}, K_m^{PEP} = 4.53 ± 0.59 mM and k_{cat}/K_m^{PEP} = 0.10 × 10^5 ± 1.08 × 10^3 M^{-1}s^{-1}; (B) Markers represent data for *Panicum pygmaeum* PEPC, the line is characterised by the parameters k_{cat} = 65.59 ± 1.26 s^{-1}, K_m^{PEP} = 0.13 ± 0.01 mM and k_{cat}/K_m^{PEP} = 5.01 × 10^5 ± 2.44 × 10^4 M^{-1}s^{-1}; (C) Markers represent experimental data for *Flaveria trinervia* PEPC, the line is characterised by the parameters k_{cat} = 47.99 ± 1.21 s^{-1}, K_m^{PEP} = 0.60 ± 0.05 mM, and k_{cat}/K_m^{PEP} = 0.79 × 10^5 ± 5.43 × 10^3 M^{-1}s^{-1}; (D) Markers represent experimental data for *Flaveria pringlei* PEPC, the line is characterised by the parameters k_{cat} = 52.65 ± 1.37 s^{-1}, K_m^{PEP} = 0.06 ± 0.01 mM and k_{cat}/K_m^{PEP} = 9.35 × 10^5 ± 8.49 × 10^4 M^{-1}s^{-1}.

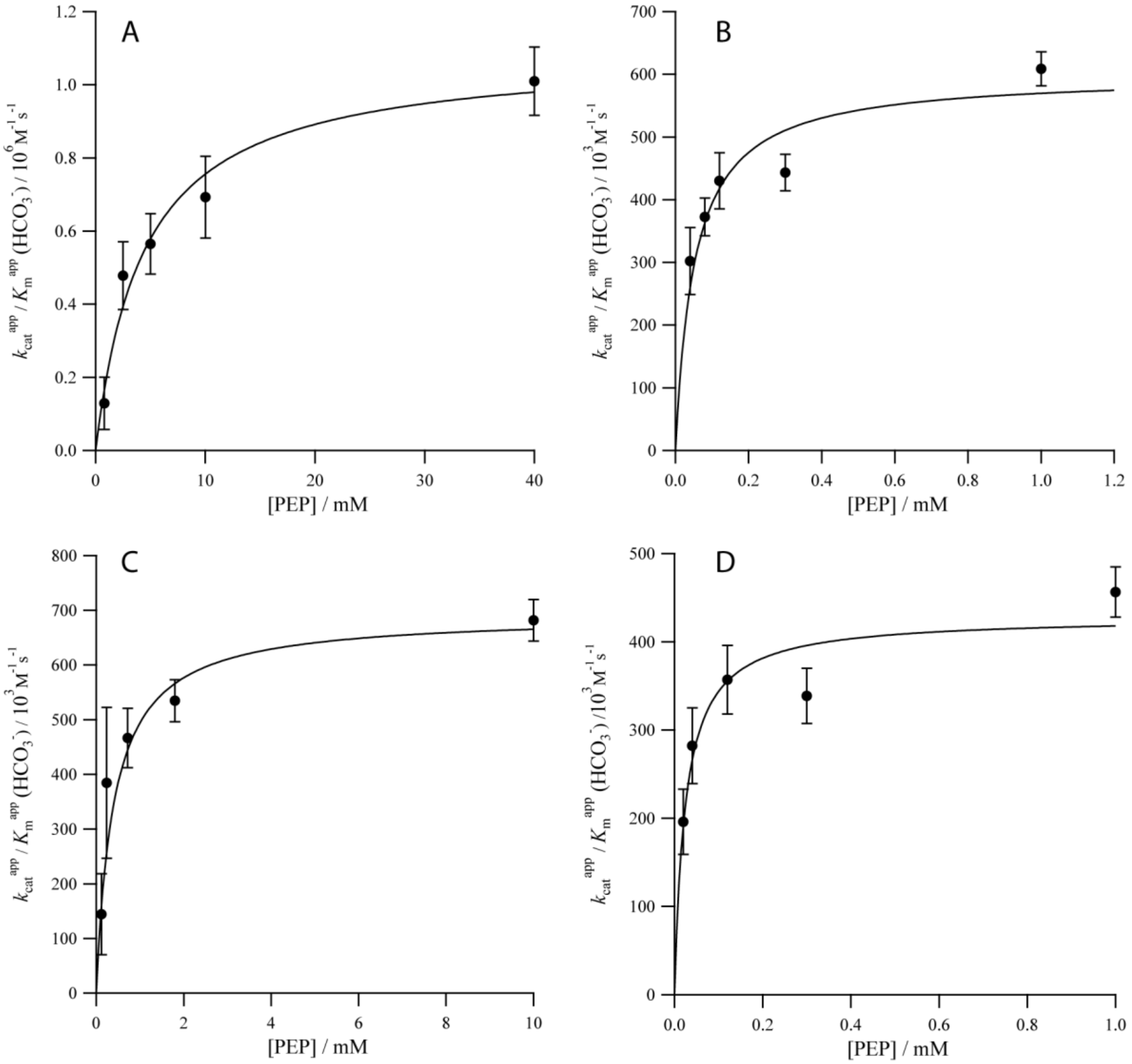

Supplementary Figure 4. Secondary analysis of bicarbonate Michaelis-Menten behaviour ($\boldsymbol{k_{cat}^{app}/K_m^{app}}$ vs PEP). Secondary plot of the $k_{cat}^{app}/K_m^{app\ HCO3-}$ parameter from fitting data shown in Figure 1, the lines are described by equation 2 and error bars represent standard errors. Markers represent the $k_{cat}^{app}/K_m^{app\ HCO3-}$ for PEPC from (A) *Panicum queenslandicum*, the line is characterised by the parameters K_i^{PEP} = 4.39 ± 1.10 mM and $k_{cat}/K_m^{HCO3-} = 1.09 \times 10^6 \pm 8.88 \times 10^4$ $M^{-1}s^{-1}$; (B) *Panicum pygmaeum*, the line is characterised by the parameters K_i^{PEP} = 0.02 ± 0.01 mM and $k_{cat}/K_m^{HCO3-} = 0.60 \times 10^6 \pm 2.93 \times 10^4$ $M^{-1}s^{-1}$; (C) *Flaveria trinervia*, the line is characterised by the parameters K_i^{PEP} = 0.40 ± 0.13 mM and $k_{cat}/K_m^{\ HCO3-} = 0.69 \times 10^6 \pm 4.17\times 10^4$ $M^{-1}s^{-1}$. (D) *Flaveria pringlei*, the line is characterised by the parameters K_i^{PEP} = 0.02 ± 0.01 mM and $k_{cat}/K_m^{HCO3-} = 0.44 \times 10^6 \pm 2.17 \times 10^4$ $M^{-1}s^{-1}$.

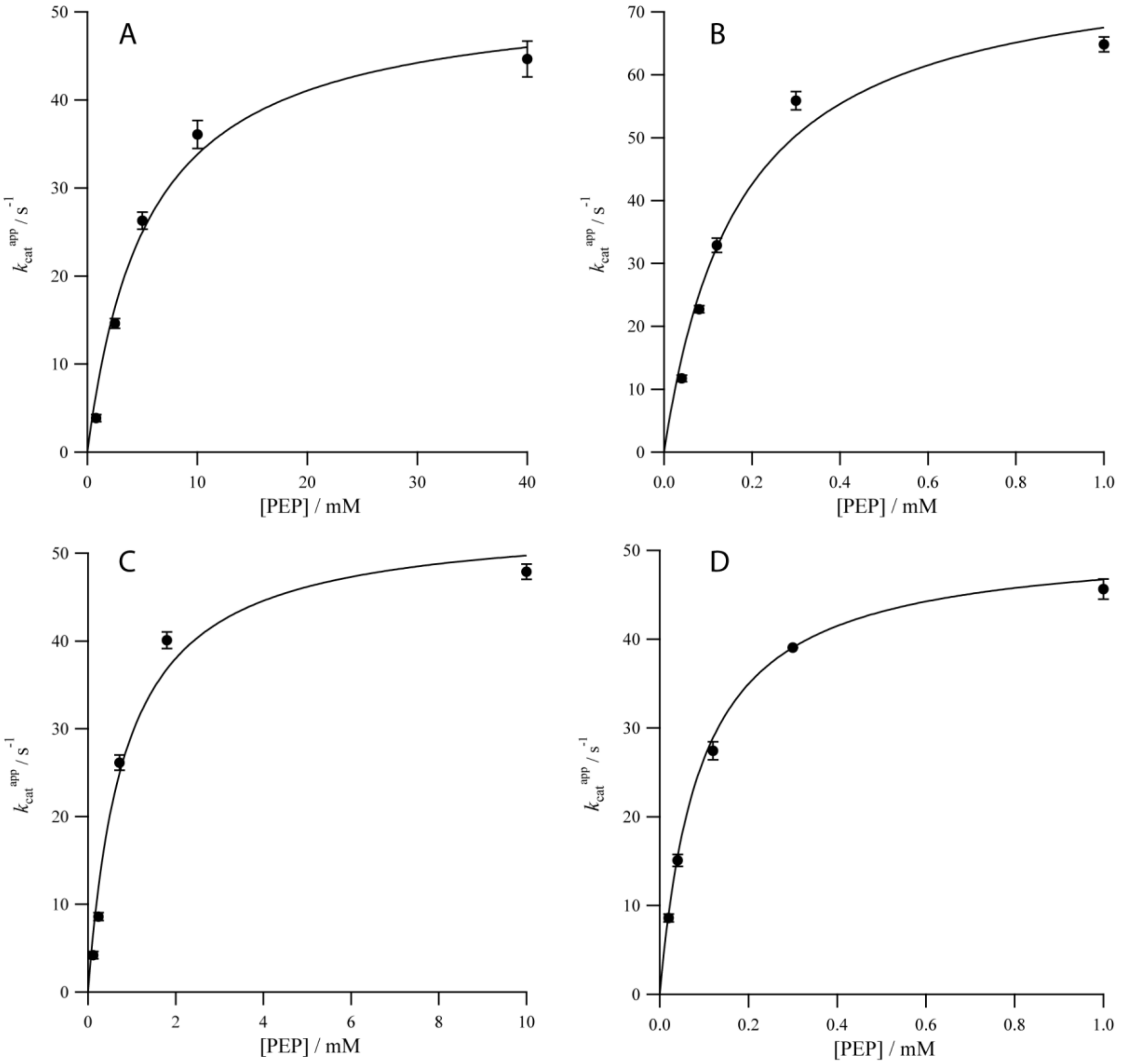

Supplementary Figure 5. Secondary analysis of bicarbonate Michaelis-Menten behaviour (k_{cat}^{app} vs PEP). Secondary plot of the k_{cat}^{app} parameter from fitting data shown in Figure 1. The line described by equation 2, error bars represent standard errors from fit of lines. Markers represent the k_{cat}^{app} parameter for PEPC from (A) *Panicum queenslandicum*, the line is characterised by the parameters k_{cat} = 52.25 ± 3.72 s^{-1} K_m^{PEP} = 5.46 ± 1.12 mM; (B) *Panicum pygmaeum*, the line is characterised by the parameters k_{cat} = 79.06 ± 6.64 s^{-1} and K_m^{PEP} = 0.17 ± 0.04 mM; (C) *Flaveria trinervia*, the line is characterised by the parameters k_{cat} = 53.89 ± 4.12 s^{-1} and K_m^{PEP} = 0.84 ± 0.02 mM; (D) *Flaveria pringlei*, the line is characterised by the parameters k_{cat} = 51.01 ± 0.05 s^{-1}, K_m^{PEP} = 0.08 ± 0.01 mM.

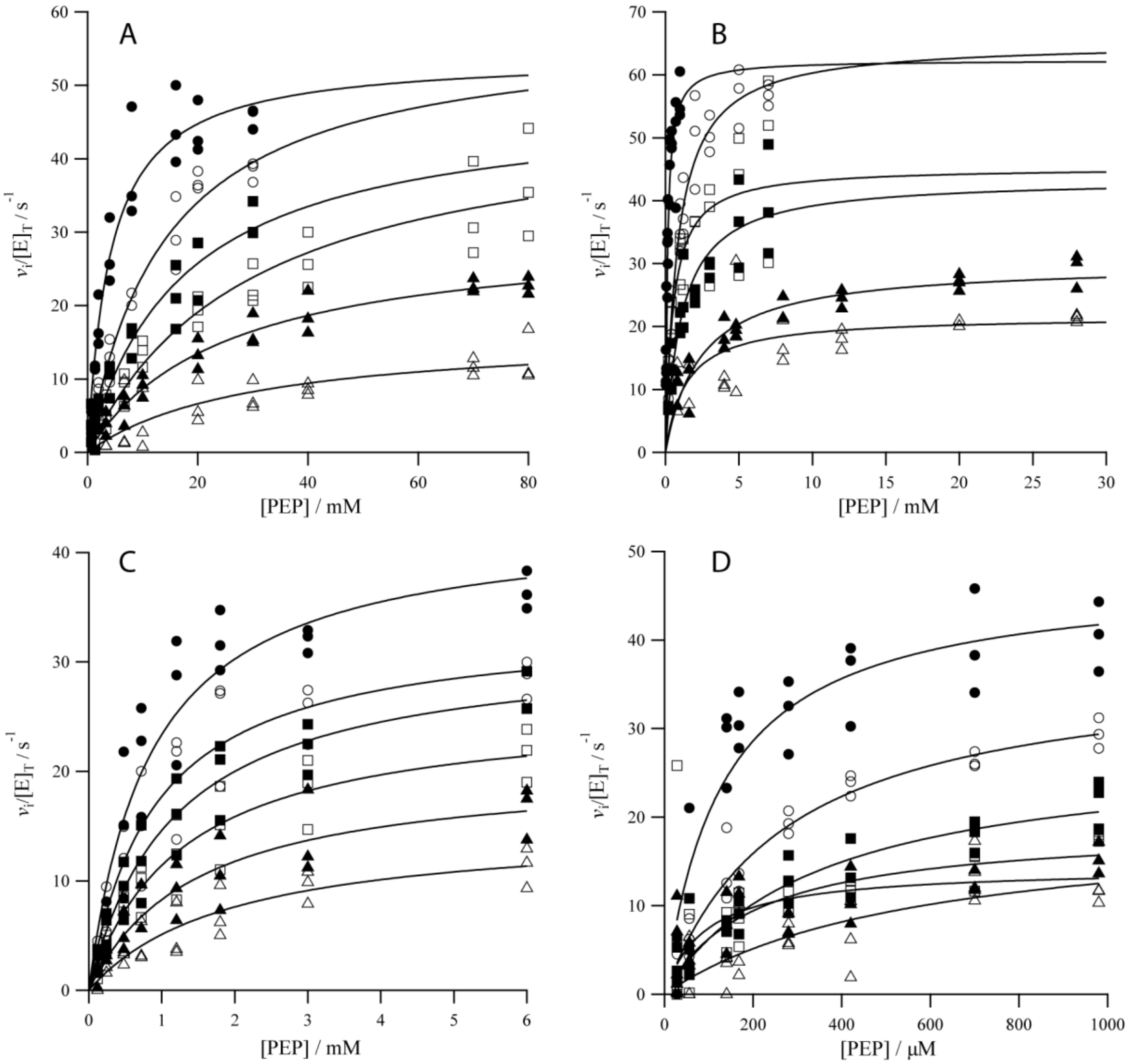

Supplementary Figure 6. Primary plots of Malate Inhibition Assays (v_i vs PEP). Assays conditions were 50 mM Tricine.KOH pH 8.0, 10 mM $MgCl_2$, 0.2 mM NADH, 0.01 Uμl^{-1} malate dehydrogenase, PEP concentration as shown on the axis. Lines are described by equation one. Assays were repeated (n = 3) for each point. Markers (in order of increasing malate: ●, ○, ■, □, ▲, △) show individual data points for the following PEPC with enzyme concentration in brackets (A) *P. queenslandicum* (10 nM), with 0, 8 mM, 16 mM, 32 mM, 60 mM and 120 mM malate; (B) *P. pygmaeum* (5 nM), with 0, 4 mM, 12 mM, 24 mM, 32 mM and 60 mM malate; (C) *F. trinervia* (10 nM), with 0, 8 mM, 16 mM, 32 mM, 60 mM and 120 mM malate; (D) *F. pringlei* (5 nM), with 0, 4 mM, 12 mM, 24 mM, 32 mM and 60 mM malate.

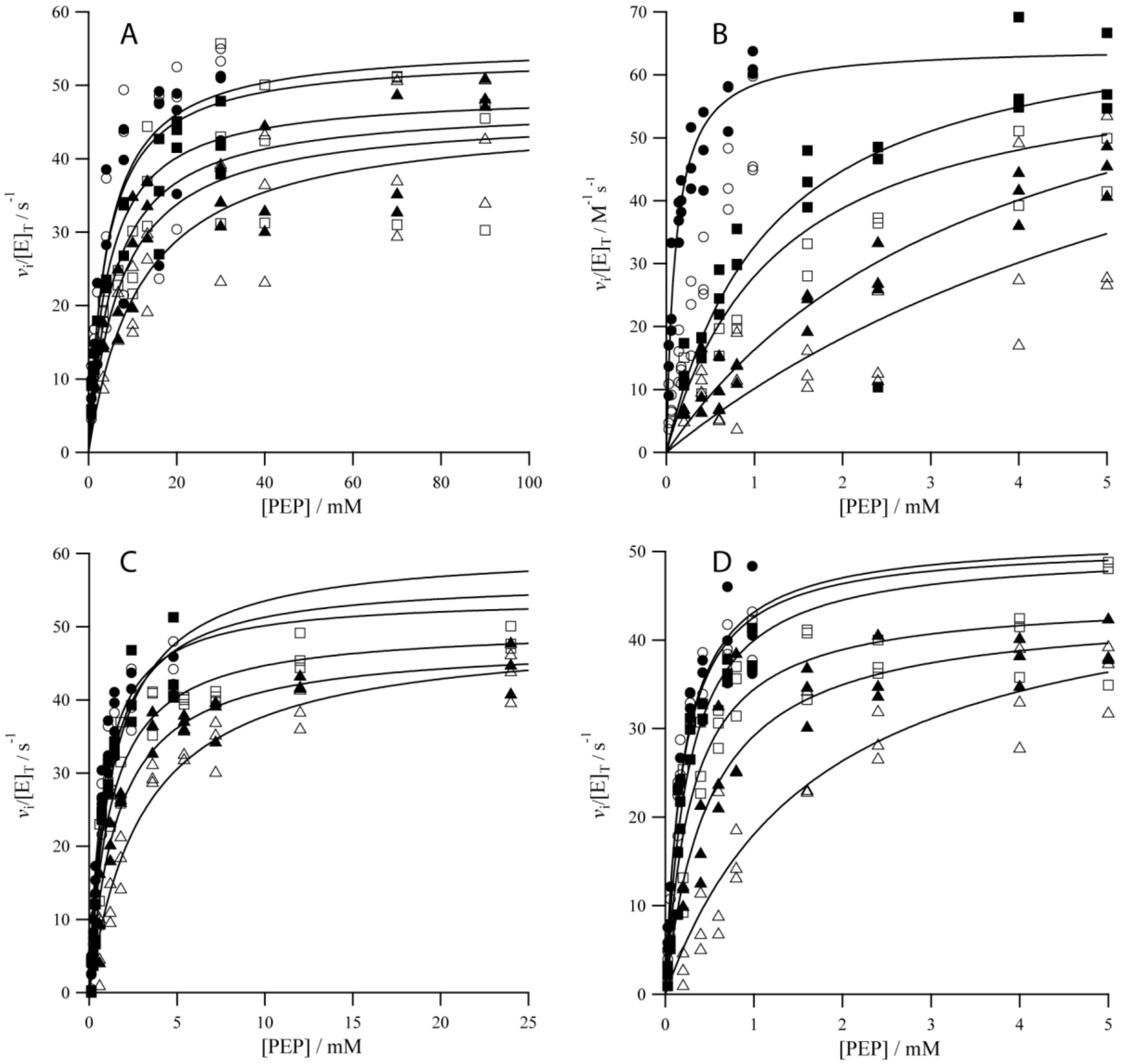

Supplementary Figure 7. Primary plots of Michaelis-Menten Aspartate Inhibition Assays (v_i vs PEP). Assays conditions were 50 mM Tricine.KOH pH 8.0, 10 mM $MgCl_2$, 0.2 mM NADH, 0.01 Uμl^{-1} malate dehydrogenase, PEP concentration as shown. Lines are described by equation one. Assays were repeated (n = 3) for each point. Markers (in order of increasing aspartate: ●, ○, ■, □, ▲, △) show individual data points for the following PEPC with enzyme concentration in brackets (A) *P. queenslandicum* (10 nM) with 0, 8 mM, 16 mM, 32 mM, 60 mM and 120 mM aspartate, 60 mM and 120 mM aspartate; (B) *P. pygmaeum* (5 nM) with 0, 8 mM, 16 mM, 32 mM; (C) *F. trinervia* (10 nM) with 0, 8 mM, 16 mM, 32 mM, 60 mM and 120 mM aspartate; (B) *P. pygmaeum* (5 nM) with 0, 8 mM, 16 mM, 32 mM; (D) *F. pringlei* (5 nM), performed in the presence of 0, 8 mM, 16 mM, 32 mM, 60 mM and 120 mM aspartate.

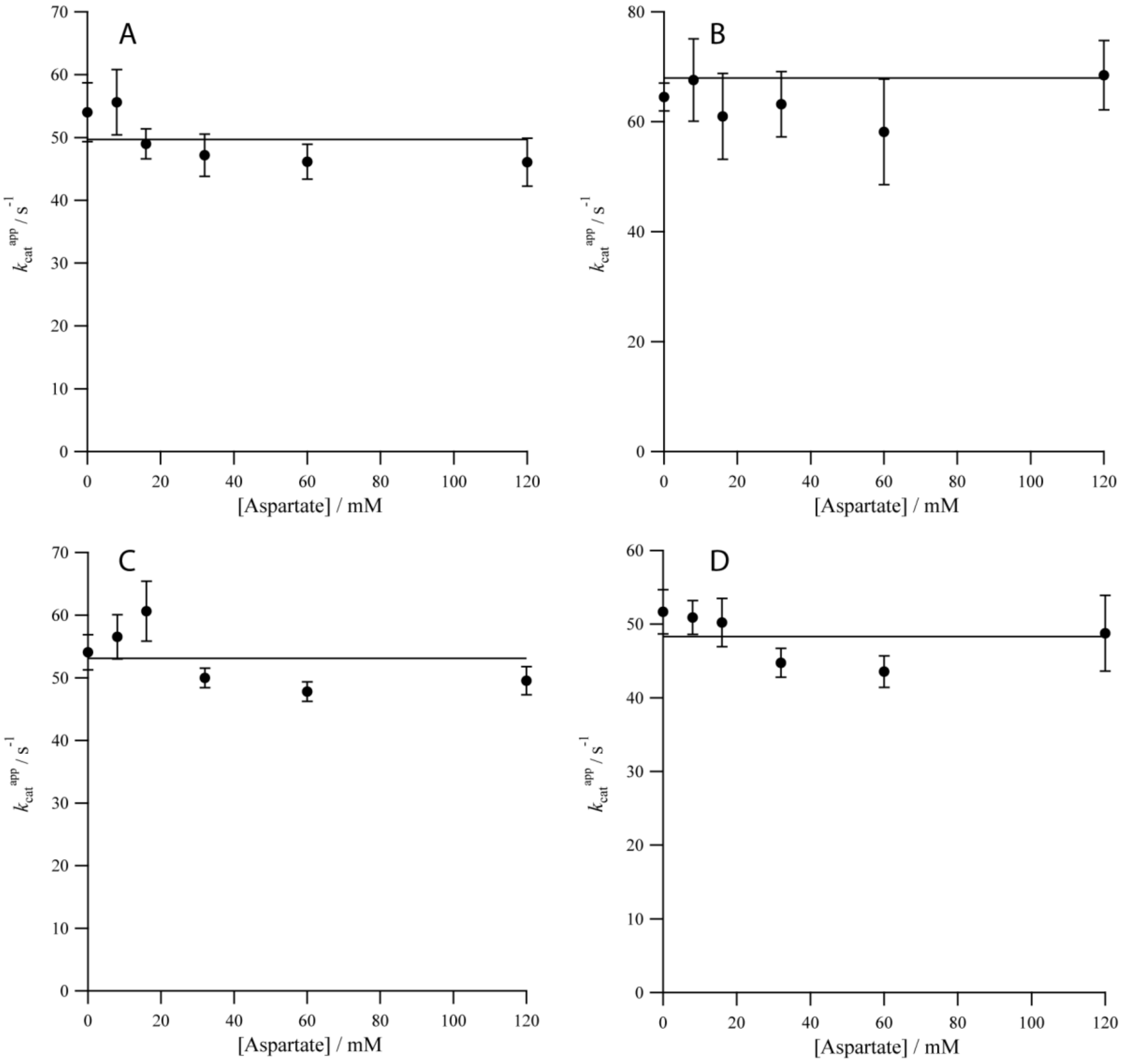

Supplementary Figure 8. Secondary plots of Aspartate Inhibition (k_{cat}^{app} vs Aspartate). Assays conditions were 50 mM Tricine.KOH pH 8.0, 10 mM $MgCl_2$, 0.2 mM NADH, 0.01 Uμl^{-1} malate dehydrogenase, in the presence of varied aspartate (0-120 mM). Lines are illustrative and error bars represent standard errors. Secondary plot of k_{cat}^{app} against aspartate concentration for PEPC from (A) *P. queenslandicum*, (B) *P. pygmaeum*, (C) *F. trinervia*, and (D) *F. pringlei*.

Supplementary Table 1 Summary of primers used in this study. Primers were used for cloning and sequencing.

Primer	Sequence
FlvFor1B	TACTTCCAATCCAATGCAATGGCTAACCGGAAT
FlvRev1B	TTATCCACTTCCAATGTTATTACTAACCGGTGTTCTGC
Flav_1303_Seq_For	AGACAAGTGTCGACTT
Flav_1832_Seq_Rev	TTGTAGAGCTGCCATG
PquFor1B	GACGACGACAAGATGGCGTCCTCCGAGCGCCACC
PquRev1B	GAGGAGAAGCCCGGTTAGCCCGTGTTCTGCATGCC
PpyFor1B	TACTTCCAATCCAATGCAATGGCAAGCAG
PpyRev1B	TTATCCACTTCCAATGTTATTATTAACCGGTATTC
Pqu_1323_Seq_For	CGTGAAGCTGGACAT
Pqu_1752_Seq_Rev	ATGACCTGCTGCTTG
Ppy_1291_Seq_For	GATGGTAGTCTGCTGG
Ppy_1791_Seq_Rev	GCTATCGCTATAACCA
T7 Promotor	TAATACGACTCACTATAGGG
T7 Terminator	GCTAGTTATTGCTCAGCGG
